# Defining vitamin D status using multi-metabolite mathematical modelling: A pregnancy perspective

**DOI:** 10.1016/j.jsbmb.2019.03.024

**Published:** 2019-03-26

**Authors:** C.H.L. Beentjes, J.P. Taylor-King, A. Bayani, C.N. Davis, J.L. Dunster, S. Jabbari, G.R. Mirams, C. Jenkinson, M.D. Kilby, M. Hewison, J.A. Tamblyn

**Affiliations:** a Mathematical Institute, University of Oxford, Oxford, UK; b Department of Chemical Engineering and Biotechnology, University of Cambridge, Cambridge, CB3 OAS, UK; c Institute of Molecular Systems Biology, Department of Biology, ETHZ, CH-8093, Zurich, Switzerland; d Department of Physics and Mathematics, School of Science and Technology, Nottingham Trent University, Nottingham, UK; e MathSys CDT, Mathematics Institute, University of Warwick, Coventry, UK; f Institute for Cardiovascular and Metabolic Research, University of Reading, UK; g School of Mathematics and Institute of Microbiology and Infection, University of Birmingham, Birmingham, UK; h Centre for Mathematical Medicine & Biology, Mathematical Sciences, University of Nottingham, UK; i Institute of Metabolism and Systems Research, College of Medical and Dental Sciences, University of Birmingham, Birmingham, UK; j Centre for Women’s & Newborn Health, Birmingham Health Partners, Birmingham Women’s & Children’s Foundation Hospital, Edgbaston, Birmingham, UK; k Centre for Endocrinology, Diabetes and Metabolism, Birmingham Health Partners, Birmingham, UK; l Fetal Medicine Centre, Birmingham Women’s & Children’s Foundation Trust, Edgbaston, Birmingham, UK

**Keywords:** Vitamin D, Pregnancy, Preeclampsia, Metabolome, Mathematical model

## Abstract

Vitamin D deficiency is linked to adverse pregnancy outcomes such as pre-eclampsia (PET) but remains defined by serum measurement of 25-hydroxyvitamin D3 (25(OH)D3) alone. To identify broader changes in vitamin D metabolism during normal and PET pregnancies we developed a relatively simple but fully parametrised mathematical model of the vitamin D metabolic pathway. The data used for parametrisation were serum vitamin D metabolites analysed for a cross-sectional group of women (n = 88); including normal pregnant women at 1 st (NP1, n = 25) and 3rd trimester (NP3, n = 21) and pregnant women with PET (n = 22), as well as non-pregnant female controls (n = 20). To account for the effects various metabolites have upon each other, data were analysed using an ordinary differential equation model of the vitamin D reaction network. Information obtained from the model was then also applied to serum vitamin D metabolome data (n = 50) obtained from a 2nd trimester pregnancy cohort, of which 25 prospectively developed PET.

Statistical analysis of the data alone showed no significant difference between NP3 and PET for serum 25(OH) D3 and 24,25(OH)_2_D3 concentrations. Conversely, a statistical analysis informed by the reaction network model revealed that a better indicator of PET is the ratios of vitamin D metabolites in late pregnancy. Assessing the potential predicative value, no significant difference between NP3 and PET cases at 15 weeks gestation was found. Mathematical modelling offers a novel strategy for defining the impact of vitamin D metabolism on human health. This is particularly relevant within the context of pregnancy, where major changes in vitamin D metabolism occur across gestation, and dysregulated metabolism is evidenced in women with established PET.

## 1 Introduction

Sub-optimal serum levels of 25-hydroxyvitamin D3 (25(OH)D3) appear to be particularly prevalent in pregnant women [1,2], prompting association studies linking maternal 25(OH)D3 status and adverse events of pregnancy [3]. These include pre-eclampsia (PET) [4–6], small for gestational age babies [7], bacterial vaginosis [8], and gestational diabetes mellitus [9,10]. However, in recent years a much broader role for vitamin D in human pregnancy has been proposed, incorporating effects on female reproductive health [11,12], male reproductive health [11,13], fetal development [14,15], fetal programming [16], and offspring health [17,18]. In humans, vitamin D supplementation of pregnant women has been carried out to investigate effects on preterm birth [19] and neonatal health [18].

It is still unclear whether vitamin D-deficiency during pregnancy is simply a manifestation of the broader prevalence of low vitamin D status in populations across the globe, or whether this reflects a normal physiological drop in 25(OH)D3 concentrations during pregnancy, or if pregnancy is a stress test which can exacerbate and unmask pathological vitamin D deficiency. Whatever the case, it is important to recognise that, to date, studies have relied on measurement of a single parameter to define optimal vitamin D in pregnancy – namely serum levels of 25(OH)D3, the major circulating form of vitamin D. We have hypothesized that measurement of total serum concentrations of 25(OH)D3 alone provides only a limited view of vitamin D status, particularly in pregnancy where significant changes in vitamin D physiology occur from early gestation [20]. This includes changes in the serum carrier vitamin D binding protein (DBP) during pregnancy [21] which may influence the bioavailability of unbound or DBP-bound 25(OH)D3 [22].

During pregnancy there is also a dramatic rise in maternal circulating concentrations of the active hormonal form of vitamin D, 1,25-dihydroxyvitamin D (1,25(OH)_2_D3) from the 1 st trimester of gestation [23]. Thus, the impact of vitamin D during pregnancy may be better defined by analysis of multiple serum vitamin D metabolites – the vitamin D metabolome. In women with PET we have previously reported significant dysregulation of multiple vitamin D metabolic pathways in the 3^rd^ trimester of pregnancy, with decreased serum 1,25(OH)_2_D3 levels and enhanced catabolic 24,25-dihydroxyvitamin D3 (24,25(OH)_2_D3) and 3-epi-25(OH)D3 concentrations observed for women with PET [20]. Conversely, in a study of pregnant women in the 1^st^ trimester of pregnancy who subsequently progressed to normal or PET pregnancies we did not observe any significant variations in serum vitamin D metabolites between normal and PET pregnancies [24]. The aim of the current study was to develop a mathematical model of the serum vitamin D metabolome to provide an improved statistical analysis for screening for adverse events in pregnancy, such as PET, by identifying markers of vitamin D function and disease based on analysis of multiple vitamin D metabolites. Specifically, we aimed to detect a signal within vitamin D metabolite data recorded both in early (1st trimester) and late (3rd trimester) pregnancy, to allow classification of cases into high and low risk of PET. The study found that, rather than considering individual metabolite levels, inspection of specific ratios of these metabolites (as determined by the mathematical model) was a more informative predictive measure. Implementing data from a larger cohort of patients will transform this study from proof of concept into a strong predictive screening tool for PET. Though we have utilised data specifically with pregnancy in mind, the approach presented provides a novel analytical tool for the study of vitamin D metabolites more generally in human health and disease.

## 2 Materials and methods

### 2.1 Human pregnancy vitamin D metabolite datasets

Serum vitamin D metabolite data obtained from two distinct sets of pregnant women was utilised to ascertain the accuracy of each statistical approach (one considering individual metabolite levels, the other their ratios). The first group of women were from a cross-sectional pregnancy study which included 3 groups of pregnant women booked in the West Midlands, UK (i) first trimester uncomplicated pregnancies (8–13 weeks) (NP1; n = 25), (ii) healthy 3^rd^ trimester pregnancies (> 37 weeks) (NP3; n = 21) and (iii) third trimester pregnancies complicated by PET (PET; n = 22). A healthy non-pregnant female ‘control’ group (n = 20) was also recruited for comparative serum vitamin D analysis as described in detail previously (West Midlands, Edgbaston REC (14/WM/1146 RG_14-194 [09.12.2016 approval]) (Table 1) [20].

For the second group of pregnant women, serum samples were purchased from the SCOPE (Screening for Pregnancy Endpoints) Ireland study (n = 50) (Clinical Research Ethics Committee of the Cork Teaching Hospital: ECM5 (10) [05.02.08 approval]) [24]. As previously described, sera samples from n = 50 low-risk nulliparous pregnant women taken at 15 weeks gestation (2^nd^ trimester) were analysed. Of these, n = 25 prospectively developed PET and n = 25 were selected as normotensive controls matched for maternal age, ethnicity and body mass index (BMI) (Table 1) [24]. Vitamin D metabolites were extracted from serum for LC–MS/MS analysis and measured as described previously [20,24,25]. Serum concentrations were obtained as outlined in Supplemental Table 1 and Supplemental Table 2.

### 2.2 Analysis of serum vitamin D metabolites

Analysis of serum concentrations of vitamin D metabolites (25(OH) D3, 3-epi-25(OH)D3, 1,25(OH)_2_D3 and 24,25(OH)_2_D3) was performed using previously reported liquid chromatography-tandem mass spectrometry (LC–MS/MS) methods [20,25]. In brief, samples were prepared for analysis by protein precipitation and supported liquid-liquid extraction. Analysis of serum was performed on a Waters ACQUITY ultra performance liquid chromatography coupled to a Waters Xevo TQ-S mass spectrometer. The LC–MS/MS method was validated based on US Food and Drug Administration guidelines for analysis of these metabolites, with CVs for each vitamin D metabolite analysis is shown in Table 2.

### 2.3 Regression analysis of vitamin D metabolite data across pregnancy

Serum vitamin D metabolite data from the West Midlands healthy pregnant (NP1 and NP3) and non-pregnant cohorts were first assessed by (linear) robust regression analysis to estimate the relationship between metabolome concentrations and gestation progression. This was performed using iteratively reweighted least squares with a bi-square weighting function using the MATLAB Curve Fitting Toolbox [26]. Robust regression using iterative re-weighting and non-standard weighting functions was chosen in favour to the more standard linear least-squares regression due to its capabilities to reliably handle outliers present in the raw data.

### 2.4 Principal component analysis

We began by applying a standard statistical analysis to the data independent of the mathematical model. We examined serum vitamin D metabolites for PET and healthy patient serum data from the SCOPE data for all patients where all five metabolites were available (N = 16 cases and N = 22 controls). Each metabolite value was normalised by multiplying by a scaling factor such that the mean for each normalised metabolite became equal to one. We then ran a principal component analysis (PCA) on the normalised dataset using the MATLAB ‘PCA’ function.

### 2.5 Linear classification analysis

The task of statistically classifying metabolite data into groups of healthy normotensive and PET women in an interpretable manner can be conveniently carried out using linear support-vector machines (SVMs) [26,27]. In this approach we find, given the training data, the best decision function which maps data to one of the two possible outcomes (healthy versus PET in our case) based on a linear combination of various features of the data, e.g. concentrations of different vitamin D metabolites. Using SVMs we can select the best possible linear decision function in terms of the accuracy of its predictions.

### 2.6 Kinetic mathematical model of vitamin D function

**Figure 1:**
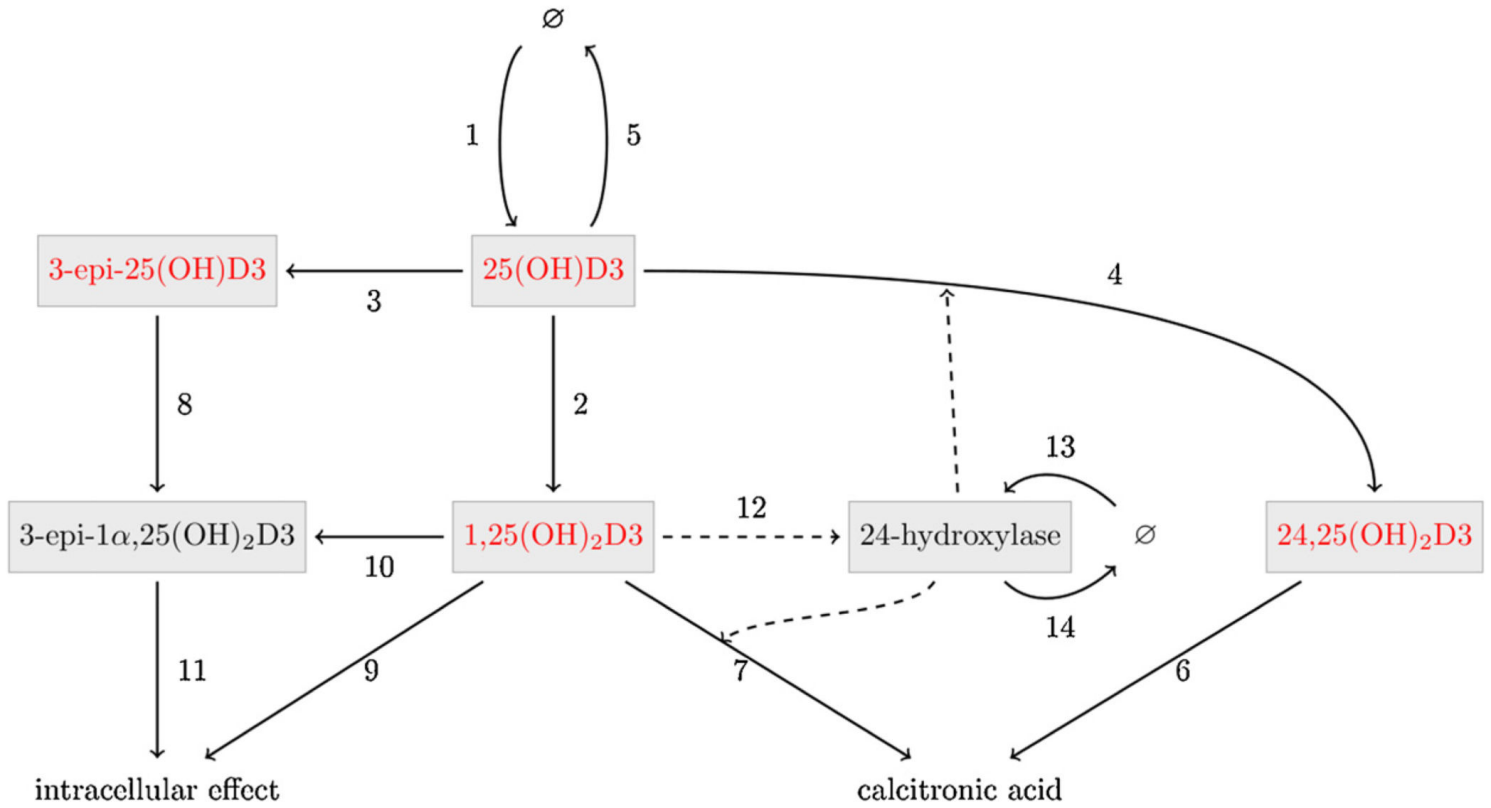
Network diagram for the kinetic model of vitamin D metabolism. Reaction network structure for vitamin D metabolome model, including the metabolites 25-hydroxyvitamin D3 (25(OH)D3); C3-epimer hydroxyvitamin D3 (3-epi-25(OH)D3); 1,25-dihydroxyvitamin D3 (1,25(OH)_2_D3); 24,25-dihydroxyvitamin D3 (24,25(OH)_2_D3); C3-epimer 1,25-hydroxyvitamin D3 (3-epi-1α,25(OH)_2_D3); and the key enzyme vitamin D-24-hydroxylase (24-hydroxylase). Black arrows depict conversion steps and dotted lines indicate enzymatic interactions, numbers are defined in Table 4. Serum data was only available for the metabolites in red.

To account for the effects that vitamin D metabolites have on each other, initially the full chemical reaction network was modelled as a system of ordinary differential equations (in the next section this was reduced to a simpler but equally informative model that can be fully parametrised). As outlined in Table 3 and Fig. 1, variables were used to design a mathematical kinetic model for vitamin D physiology that incorporated the main cellular features of vitamin D metabolism and function; active, inactive and epimerised forms of vitamin D, as well as the key catabolic enzyme 24-hydroxylase [33,34]. These cellular variables were then used as a basis to model circulating vitamin D metabolites.

The metabolite network for vitamin D (Fig. 1) was mathematically interrogated by constructing a set of ordinary differential equations (ODEs), depicted in Tables 4 and 5, describing the time-dependent behaviour of vitamin D metabolites in the metabolite network. Based on saturation kinetics for vitamin D metabolism [28], we assumed that the conversion of 25(OH)D3 to 1,25(OH)_2_D3 follows Michaelis-Menten kinetics. For simplicity, and to facilitate model parametrisation, all other reactions were assumed to obey mass action kinetics (i.e. either they do not saturate or they do not saturate at the levels present in our data).

### 2.7 A reduced mathematical model of vitamin D function

**Figure 2:**
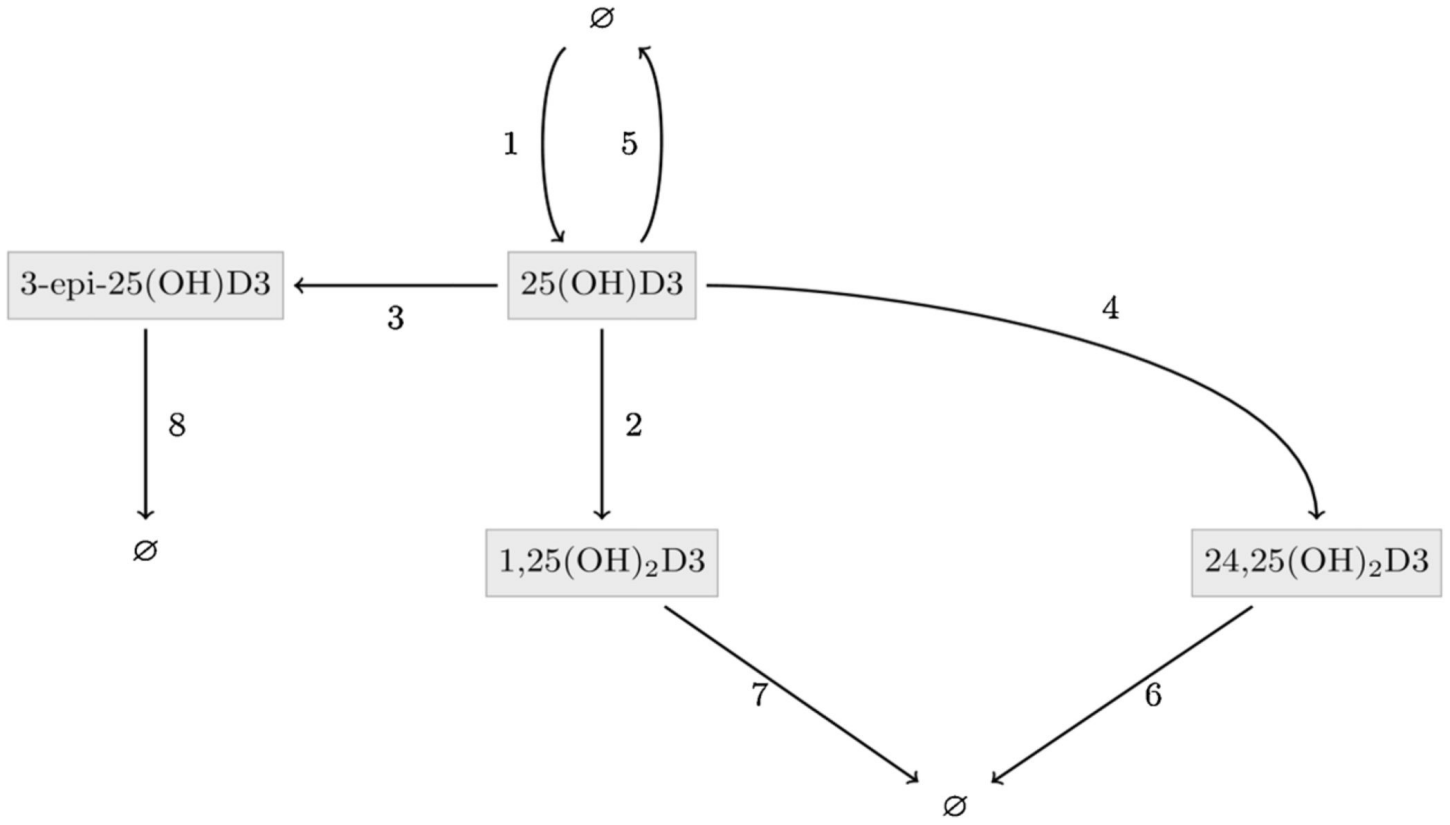
Network diagram of the reduced kinetic model for vitamin D metabolism. The network only contains chemicals with measured concentrations, 25-hydroxyvitamin D3 (25(OH)D3); C3-epimer 25-hydroxyvitamin D3 (3-epi-25(OH)D3); 1,25-dihydroxyvitamin D3 (1,25(OH)_2_D3) and 24,25-dihydroxyvitamin D3 (24,25(OH)_2_D3). Black arrows depict conversion steps, and their numbers are defined in Table 6.

As shown in Table 3, serum concentrations of 3-epi-1a,25(OH)_2_D3 and 24-hydroxylase enzyme activity were not measured in the current study. A reduced mathematical model of vitamin D metabolism was therefore designed by applying simplifying assumptions into the complete model given in Table 5, incorporating solely 25(OH)D3; 1,25(OH)_2_D3; 24,25(OH)_2_D3 and 3-epi-25(OH)D3; and their effective mutual interactions (Fig. 2). Firstly, we assume that levels of 24-hy-droxylase are relatively constant so that we can assume conversion of 25(OH)D3 into 24,25(OH)_2_D3 and loss of 1,25(OH)_2_D3 from the system occur at a constant rate. Secondly, as we have no information regarding 3-epi-1a,25(OH)_2_D3, we can simply consider loss of 3-epi-25(OH)D3 and 1,25(OH)_2_D3 from the system to also capture their loss through conversion into 3-epi-1a,25(OH)_2_D3. Thus, these simplifying assumptions (that have the benefit of facilitating complete model parameterisation) should not have any untoward effect on overall data. The resulting reduced network has 8 unknown reaction constants and kinetic rate equations (see Tables 6 and 7) and can be described by a system of four ODEs (see Table 7). The equations can be fully parameterised (see Supplementary Information), and the steady states readily obtained (Table 8). These steady states are used to inform the statistical analysis in Section 3.4.

## 3 Results

### 3.1 Regression analysis of vitamin D metabolite data across pregnancy

**Figure 3:**
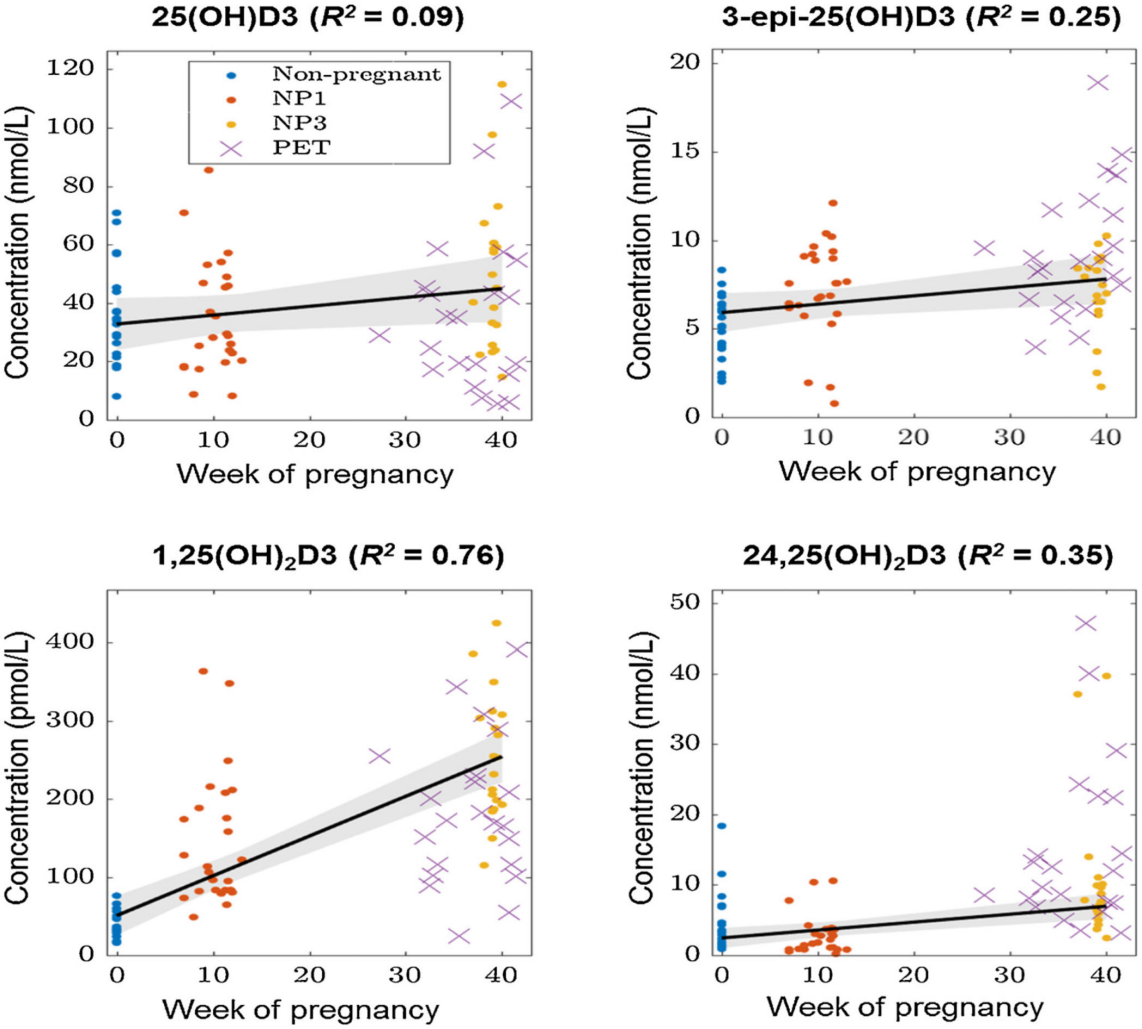
Regression analysis of serum vitamin D metabolites across normal pregnancy. Serum concentrations of 25-hydroxyvitamin D3 (25(OH)D3), C3-epimer 25-hydroxyvitamin D3 (3-epi-25(OH)D3), 1,25-dihydroxyvitamin D3 (1,25(OH)_2_D3), 24,25-dihydroxyvitamin D3 (24,25(OH)_2_D3) according to week of pregnancy. Grey areas show the 95% confidence bands around fitted regression lines (black lines solid lines) and *R^2^* values for each regression are reported under their respective graphs. PET samples (3^rd^ trimester) were not included in the regression but are shown for reference.

Consistent with previous single metabolite data [24], regression analysis of data from the first group of pregnant women (West Mid-lands) showed increased serum concentrations of 1,25(OH)_2_D3 across normal pregnancy, but also revealed trends towards increased concentrations of 25(OH)D3, 3-epi-25(OH)D3, and 24,25(OH)_2_D3 from 1st to 3rd trimester (Fig. 3). Data for women with PET were not included in the regression analysis but are shown for reference, providing further evidence for dysregulation of vitamin D metabolism in PET relative to healthy 3rd trimester pregnancies.

### 3.2 Principal component analysis (PCA)

Using strategies outlined in Section 2.4, PCA analysis was carried out to further interrogate serum vitamin D metabolite data from the West Midlands group. The first two components (PCA1 and PCA2) explained 74% of data variance but did not appear to separate PET and healthy 3rd trimester pregnancies (Supplemental Fig. 1). It was possible that another classifier could distinguish the normal and PET groups based on the remaining variation, or that a non-linear projection was required. To investigate these possibilities, we ran multiple classification algorithms using the MATLAB Classification Toolbox [32], however no strong classifiers were identified. We therefore utilised to two linear classifier analyses: one independent from the mathematical model and one informed by the mathematical model.

### 3.3 Strategy 1: linear classifier analysis of metabolite data

**Figure 4:**
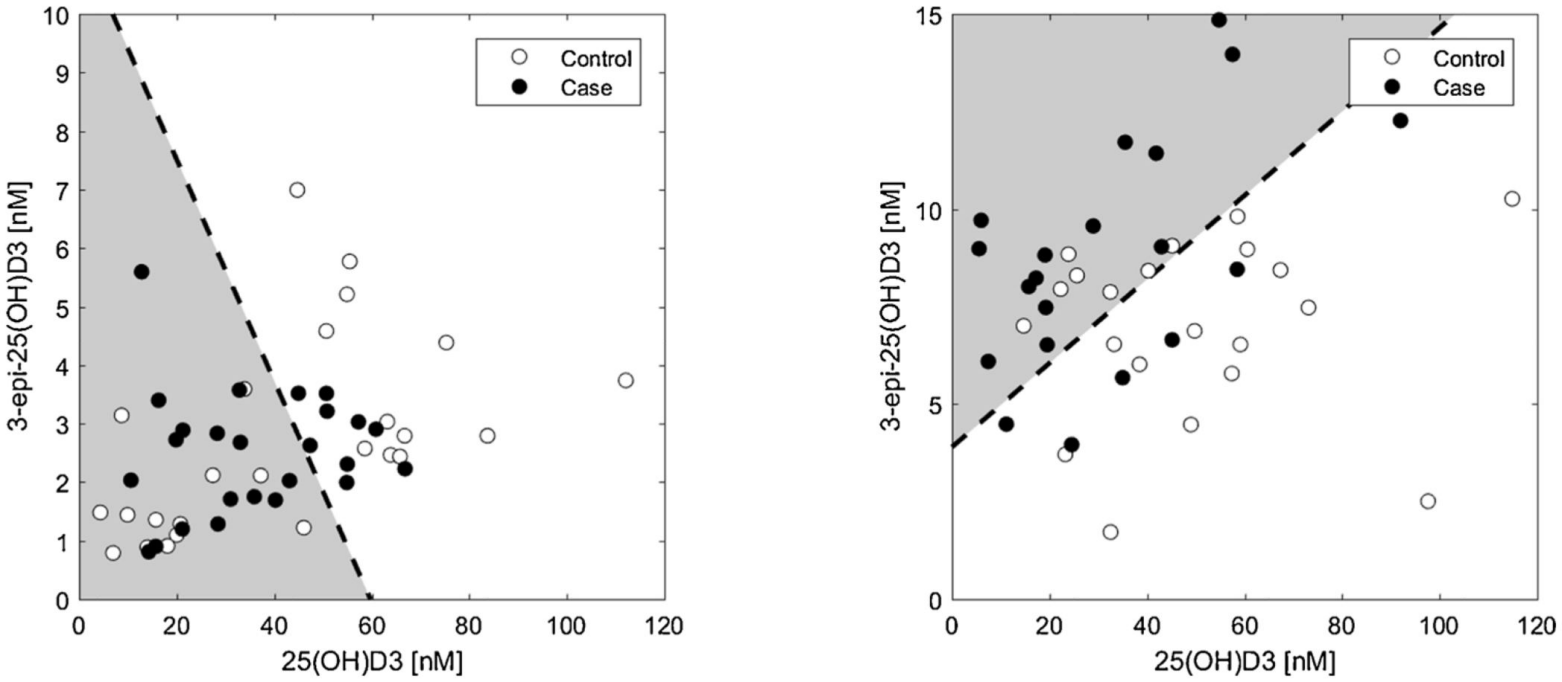
A two-dimensional linear classifier (dotted line) incorporating serum 3-epi-25(OH)D3 (y-axis) and 25(OH)D3 [nM] (x-axis) concentrations for the SCOPE (15 weeks of pregnancy) (left) and West Midlands (third trimester) data (right). The linear classifier classes serum data in the grey section as PET case and the remainder as being healthy pregnancies. False-positives and false-negatives are clearly visible and lead to a classification accuracy of only 56% (left) and 68% (right).

Using data from the West Midlands group and 2nd trimester data from the SCOPE group we investigated a possible linear classifier strategy for defining normal healthy and PET pregnancies based on serum vitamin D metabolites. This approach aimed to delineate control/healthy normotensive and case/PET pregnant women by using a data dividing plane, as illustrated in Fig. 4. The dividing plane found is the best possible given the data in this study. A one-dimensional linear classifier specifies a single criterion for a single metabolite to predict whether the data represents control or case. A two-dimensional classifier, as illustrated in Fig. 4, sets combined criterion for two metabolites. For example, the threshold for 3-epi-25(OH)D3 increases as 25(OH)D3 increases and vice versa. For a combination of more than two metabolites the linear classifier generalises to provide a criterion based on the combined metabolite data. The efficacy of the linear classifiers is determined by the accuracy of the classification (percentage of correct classifications on the data set). Only classifiers achieving significantly > than 50% accuracy were of interest.

From earlier work [24] we postulated that individual vitamin D metabolite data cannot accurately predict future risk of PET onset. As shown in Fig. 4, individual metabolite classifiers did not reach accuracies > 68%. However, combining the data of four metabolites (25(OH)D3, 3-epi-25(OH)D3, 1,25(OH)_2_D3, 24,25(OH)_2_D3) for a single and finding the best four-dimensional linear classifier increased the accuracy of distinguishing between PET and normal pregnancies to 74%, suggesting that measurement of multiple vitamin D metabolites has the potential to be a more accurate representation of vitamin D ‘status’.

### 3.4 Strategy 2: linear classifier analysis on metabolite ratios informed by the mathematical model

An alternative strategy to analyse serum vitamin D metabolite data in pregnancy is to consider metabolite ratios. Rather than calculating all possible combinations of vitamin D metabolites to form pairwise ratios, informative ratios, designated as *α*, *β* and *γ* (Table 9), were determined directly from the steady state relationships outlined in the reduced kinetic model for vitamin D metabolism ((3.1b)–(3.1d), Table 8).

Using the estimated Michaelis constant *K* (Supplementary Table 4) individual patient data were transformed (non-linearly) into three metabolite ratios (Table 9), reflecting the different major pathways in vitamin D metabolism. For example, rather than looking at isolated 3-epi-25(OH)D3 values, the concentration of epimerised 25(OH)D3 in relation to 25(OH)D3 was considered, encoded by ratio ‘*β*’. This approach appears more informative than interpretation of single 3-epi-25(OH)D3 concentrations which alone do not help explain whether an increased intake of 25(OH)D3 determines downstream vitamin D metabolite concentrations, or whether it is the epimerisation pathway that is specifically upregulated.

**Figure 5:**
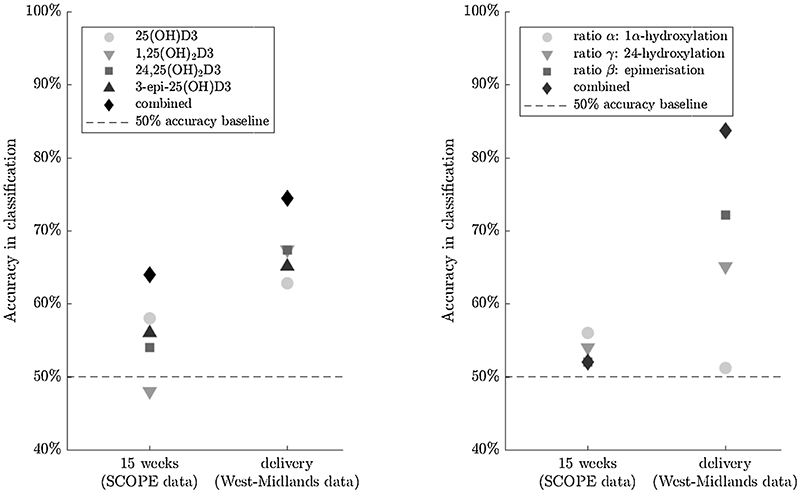
Accuracy of best linear classifiers of data. Accuracy classification is shown for 2^nd^ trimester (SCOPE) and 3^rd^ trimester (West Midlands) data. A. Based on data for individual vitamin D metabolites, and combined vitamin D metabolites (diamonds). B. based on individual metabolite or combined metabolite ratios (diamonds). Reference baseline accuracy of 50% is indicated by dotted lines.

The effect of transforming metabolite data into ratios before finding the best linear classifier was also assessed. This revealed significant improvement in the accuracy of classifiers. For example, solely utilising the 3rd trimester epimerisation ratio (3.2b) as the linear classifier, results in an accuracy of 72% in delineating between healthy and PET pregnancies. Combining metabolite ratios together resulted in an accuracy of 84% for the West Midlands 3rd trimester data. The high accuracy for the linear classifier based on the epimerisation ratio, β, alone suggests that changes in the vitamin D epimerisation pathway may be a marker of PET. Conversely, as illustrated in Fig. 5, the combined metabolite ratio analysis did not improve the overall accuracy of classification in the 2nd trimester SCOPE data.

## 4 Discussion

Vitamin D-deficiency has been linked to the pregnancy disorder PET, a condition that can increase morbidity and mortality in pregnant women, and their unborn and newborn babies. To date, studies of vitamin D and PET have focused on serum measurement of a single vitamin D metabolite, 25(OH)D3 despite the fact that pregnancy is characterised by distinct changes in vitamin D metabolism [29,30]. The metabolic pathway for vitamin D is relatively well-defined and multiple vitamin D metabolites are measurable from serum. Using data from pregnant and non-pregnant women, where concentrations of four of these metabolites have been measured, the principal aim of this study was to use a combination of data analysis and mathematical modelling to identify differences in vitamin D metabolism between normal and PET pregnancies. In particular, the data from 2^nd^ trimester pregnant women in the SCOPE study investigated to determine whether the models we used could accurately classify healthy normotensive (control) and PET (case) pregnant women prior to the onset of PET symptoms based upon early 2nd trimester vitamin D metabolite data.

Mathematical modelling of metabolic networks can often lead to large numbers of ordinary differential equations governed by many parameters that are often neither measurable nor straightforward to estimate from experimental data. To address this, data from 3^rd^ trimester pregnant women were utilised to develop a kinetic model of vitamin D metabolism incorporating 25(OH)D3, 3-epi-25(OH)D3, 1,25(OH)_2_D3 and 24,25(OH)_2_D3. From this we were able to reduce the full vitamin D network to include only these metabolites without disturbing the network shape surrounding them. In conjunction with literature values for half-life times this facilitated full parametrisation of the ordinary differential equation model of the reduced vitamin D network, an achievement that is rare in related mathematical models.

In the current study we compared two main modelling strategies, (i) linear classifier analysis using metabolite data and (ii) linear classifier analysis using metabolite ratios; the latter was motivated by analysis of our reduced kinetic model of vitamin D metabolism. It was shown that this permits more accurate classification of trends in vitamin D metabolism in normal and disease pregnancy compared to individual metabolite data analysis. Moreover, utilising combined metabolite ratios to reflect the interplay between the major metabolic pathways 1α-hydroxylation, epimerisation and 24-hydroxylation, a more accurate classification of 3^rd^ trimester healthy controls and PET cases was obtained.

The high accuracy for the linear classifier based on the epimerisation ratio suggests changes in the epimerisation pathway of the vitamin D metabolism could be a marker of PET. Since this was only visible in the third trimester it appears changes in vitamin D metabolism are gradual and that significant dysregulation develops as a result of PET, rather than being a precursor to disease development, although it is important to recognise that the current study involved relatively small samples sizes.

## 5 Summary and future work

The current study describes a novel strategy for understanding the differences in vitamin D metabolism between pregnant and non-pregnant women and distinguishing those pregnant women who have developed the pregnancy disorder PET. A major limitation of the current study was the small sample numbers in both the 2^nd^ trimester (SCOPE) and 3^rd^ trimester (West Midlands) studies. Future studies will aim to collect data from larger diverse cohorts of women to improve the reliability of both the statistical analyses and the model parametrisation described in the current mathematical analysis. The aim of mathematical modelling in this fashion is to generate new hypotheses for variations in vitamin D metabolism during pregnancy. In turn this may help to identify novel intervention and supplementation strategies for both normal pregnancy and PET. However, the potential applications for this mathematical modelling strategy extend far beyond pregnancy, with clear opportunities for refining our overall approach to the assessment of vitamin D related health and disease.

## Supplementary Material

Supplementary Material

## Figures and Tables

**Table 1:** Demographic summary and analysis of participants in West Midlands and SCOPE women analysed in this study. The West Midlands group includes non-pregnant female controls, normal pregnant women (n = 20) at first (NP1, n = 25) and third trimester (NP3, n = 21) and women with PET (n = 22). The SCOPE group includes PET cases (n = 25) matched to normotensive controls (n = 25) for age, ethnicity and body mass index (BMI). Total frequency (n) with percentage of total group (%), and median values with 25^th^-75^th^ interquartile range (IQR) values were calculated as stated. Statistically significant variations and post hoc test analyses are summarised; ^A^ ANOVA; ^T^ t-test * p < 0.05, ** p < 0.01, *** p < 0.001, **** p < 0.0001.

	West Midlands					SCOPE	
	Nonpregnant (n = 20)	NP1 (n = 25)	NP3 (n = 21)	PET (n = 22)	Post-hoc analysis	Normotensive controls (n = 25)	PET cases (n = 25)
**Parity:** nulliparous, total (%); multiparous, total (%)	–	–	3 (14.3%); 18 (85.7%)	18 (81.8%); 4 (18.2%)	–	25 (100.0%)	25 (100.0%)
**Maternal age**, mean; SD, year	36	27.8	33.6	28.1	^**A**^ Non-pregnant & NP1 (**); Non-	30.5	30.5
	13.5	7.3	3.8	7.3	pregnant & PET (*)	3.4	3.7
**BMI**, mean; SD, unit	–	27.8 8.1	28 5.1	27.8 6.1	ns	30.4 3.4	30.5 3.7
**Mean arterial blood pressure (MABP)**,	–	88.2	85.3	111.9	^**A**^ NP1 & PET (****); NP3 & PET	92.2	119.7****
mean; SD, unit		9.3	8.2	9	(****)	5.4	9.5
**Vitamin D supplements/day (400iu),** total (%)	0 (0%)	–	7 (33.3%)	1 (4.55%)	ns	9 (36.0)	3 (12.0)
**Positive smoking status**, total (%)	0 (0%)	–	0 (0%)	2 (9.1%)	ns	2 (8.0)	4 (16.0%)
**Gestational age at delivery,** mean; SD, week	–	10.1 1.7	39 0.7	36.7 3.9	ns	40.4 1	38.0 **** 2.2
**Birthweight,** mean; SD, grams	–		3521 397	2710 1121	^**T**^ NP3 & PET (**)	3717 522.9	3109 ** 802

**Table 2:** Accuracy and precision data for vitamin D metabolite analyses. Intra-day precision was calculated based on %RSD following the analysis of six replicates for each of the three concentration levels of each metabolite. Inter-day precision was performed over three days calculated based on %RSD following the analysis of six replicates for each of the three concentration levels of each metabolite per day for three days. Accuracy represents the percentage deviation from the true value and was calculated based on the analysis of six replicates of each concentration level for each metabolite.

Compound	Concentration (ng/mL)	Level	Precision (% RSD)		
			Intra-day	Inter-day	Accuracy (%)
**25OHD3**	0.500	Low	7.61	9.20	100.15
	5.00	Medium	10.68	7.45	91.34
	20.00	High	4.18	4.93	106.84
**3-Epi-25OHD3**	0.188	Low	8.38	11.61	103.83
	1.875	Medium	8.41	6.10	111.74
	7.500	High	3.20	5.25	97.45
**1α,25(OH)_2_D3**	0.061	Low	9.64	8.20	111.80
	0.750	Medium	3.05	5.30	99.33
	2.000	High	4.29	4.20	99.96
**23R,25(OH)_2_D3**	1.00	Low	6.46	9.62	98.02
	4.00	Medium	9.31	9.17	98.56
	8.00	High	4.49	10.85	96.71
**24R,25(OH)_2_D3**	0.500	Low	6.19	8.13	98.62
	4.00	Medium	7.22	8.26	98.47
	8.00	High	6.08	8.48	100.40
**25OHD2**	1.000	Low	10.59	9.58	96.47
	2.500	Medium	9.52	8.38	92.91
	10.00	High	14.19	9.80	99.13
**24OHD2**	0.625	Low	9.26	7.07	99.11
	1.000	Medium	7.46	6.86	108.03
	2.500	High	3.37	5.65	90.77
**3-Epi-25OHD2**	0.500	Low	11.11	8.20	114.80
	1.875	Medium	3.75	6.62	110.89
	7.500	High	0.69	3.15	100.98
**1α,25(OH)_2_D2**	0.075	Low	10.44	7.88	90.44
	0.750	Medium	4.08	7.60	93.19
	2.000	High	5.34	3.63	102.87
**1α,24(OH)_2_D2**	0.0630	Low	8.45	10.91	96.30
	0.625	Medium	4.24	5.96	102.41
	2.000	High	4.10	5.20	109.39
**7αC4**	0.375	Low	5.54	8.00	106.13
	3.750	Medium	9.81	8.71	96.07
	15.00	High	7.85	8.20	98.55

**Table 3:** Metabolite variables used in the full vitamin D kinetic model. Summary of the vitamin D metabolites and enzymes utilised in the full kinetic model with their abbreviation, functional description and measurement status included.

Metabolite/enzyme variable	Abbreviation	Description	Measured
25-hydroxyvitamin D3	25(OH)D3	storage pro-hormone vitamin D	✓
1,25-dihydroxyvitamin D3	1,25(OH)_2_D3	active vitamin D	✓
24,25 dihydroxyvitamin D3	24,25(OH)_2_D3	inactive vitamin D	✓
C3-epimer 25-hydroxyvitamin D3	3-epi-25(OH)D3	epimer of pro-hormone vitamin D	✓
C3-epimer 1,25-hydroxyvitamin D3	3-epi-1a,25(OH)_2_D3	epimer of active vitamin D	✗
vitamin D-24-hydroxylase	24-hydroxylase	catabolic enzyme	✗

**Table 4:** Reaction rate terms for the full kinetic model of vitamin D metabolism shown in Fig. 1. Reaction numbers correspond to Fig. 1. Metabolites and enzymes with abbreviations are shown in Table 3. Vitamin D metabolite concentrations and the Michaelis constant *K* are measured in nM. Non-catalytic rate constants (*k*_3_ - *k*_14_) and catalytic rate constants (*V*_7_, *V*_9_) are measured in d^−1^ and nM^−1^ d^−1^ respectively (d = days). Base production constants (*P*_25(OH)D3_ and *P*_24–hydroxylase_) and maximal enzyme rate *a* are measured in nM•d^−1^.

Reaction	Description	Rate term
1	Production of 25(OH)D3 from diet and sunlight	*P*_25(*OH*)*D*3_
2	Conversion by 1α-hydroxylase of 25(OH)D3 to 1,25(OH)_2_D3	*a*·[25(OH)D3]/(*K* + [25(*OH*) *D*3])
3	Epimerisation of 25(OH)D3 to 3-epi-25(OH)D3	*k_3_·[25(OH)D3]*
4	Enzymatic conversion by 24-hydroxylase of 25(OH)D3 to 24,25(OH)_2_D3	*V*_4_·[24 − hydroxylase]-[25(*OH*)*D*3]
5	Degradation of 25(OH)D3	*k*_5_·[25(*OH*)*D*3]
6	Conversion of 24,25(OH)_2_D3 to calcitronic acid	*k*_6_·[24,25(*OH*)_2_*D*3]
7	Enzymatic conversion by 24-hydroxylase of 1,25(OH)_2_D3 to calcitroic acid	*V*_7_·[24 − hydroxylase]· [1,25(*OH*)_2_D3]
8	Conversion by 1α-hydroxylase of 3-epi-25(OH)D3 into 3-epi-1a,25(OH)_2_D3	*k*_8_·[3 − *epi − 25(OH)D3]*
9	Vitamin D Receptor (VDR) activation	*k*_9_·[1,25(*OH*)_2_*D*3]
10	Epimerisation of 1,25(OH)_2_D3 to 3-epi-1a,25(OH)_2_D3	*k*_10_·[24,25(*OH*)_2_*D*3]
11	Vitamin D Receptor (VDR) activation	*k*_11_·[3 − *epi* − 25(*OH*)_2_*D*3]
12	Enzyme production stimulated by 1,25(OH)_2_D3	*k*_12_·[1,25(*OH*)_2_*D*3]
13	Basal enzyme production	*p*_24_–*hydroxylase*
14	Degradation of enzyme	*k*_14_·[24 − *hydroxylase]*

**Table 5:** Kinetic equations representing the vitamin D metabolism model network depicted in Fig. 1. Reaction terms *Ri* for *i* = 1, …,14 correspond to the respective rate terms as given in Table 4. Variables whose concentrations are tracked in the model are concentrations of the vitamin D metabolites 25-hydroxyvitamin D3 (25(OH)D); C3-epimer 25-hydroxyvitamin D3 (3-epi-25(OH)D); 1,25-dihydroxyvitamin D3 (1,25(OH)_2_D); 24,25-dihydroxyvitamin D3 (24,25(OH)_2_D); C3-epimer 1,25-hydroxyvitamin D3 (3-epi-1α,25(OH)_2_D); and the enzyme vitamin D-24-hydroxylase (24-hydroxylase).

d[25(OH)D3]/dt	= *R*_1_ − *R*_2_ − *R*_3_ − *R*_4_ − *R*_5_
d[1,25(*OH*)_2_D3]/dt	= *R*_2_ − *R*_7_ − *R*_9_ − *R*_10_
d[24,25(*OH*)_2_D3]/dt	= *R*_4_ − *R*_6_
d[3epi-25(OH)D3]/dt	= *R*_3_ − *R*_8_
d[3epi-1,25(*OH*)_2_D3]/dt	= *R*_8_ + *R*_10_ − *R*_11_
d[24-hydroxylase]/dt	= *R*_12_ + *R*_13_ − *R*_14_

**Table 6:** Kinetic rate terms for the reduced kinetic model of vitamin D metabolism in Fig. 2. Reaction numbers correspond to numbered interactions as shown in Fig. 2. Metabolites are listed with abbreviations in Table 3. Concentrations and Michaelis constant *K* are measured in nM. Non-catalytic rate constants (*k*_3_ - *k*_8_) are measured in d^−1^. Base production constants (P*_25(OH)D3_*) and maximal enzyme rate (*a*) are measured in nM d^−1^.

Reaction	Description	Rate term
1	Production of 25(OH)D3 from diet and sunlight	*P*_25(*OH*)*D*3_
2	Conversion by 1α-hydroxylase of 25(OH)D3 to 1,25(OH)_2_D3	*a*·[*25*(*OH*)D3]/(*K* + [*25*(*OH*)*D*3])
3	Epimerisation of 25(OH)D3 to 3-epi-25(OH)D3	*k*_3_·[25(*OH*)*D*3]
4	Conversion by 24-hydroxylase of 25(OH)D3 to 24,25(OH)_2_D3	*k*_4_·[25(*OH*)*D*3]
5	Degradation of 25(OH)D3	*k*_5_·[25(*OH*)*D*3]
6	Enzymatic conversion of 24,25(OH)_2_D3 to calcitroic acid	*k*_6_·[24,25(*OH*)_2_*D*3]
7	Effective degradation of 1,25(OH)2D3	*k*_7_·[1,25(*OH*)_2_*D*3]
8	Effective degradation of 3-epi-25(OH)D3	*k*_8_·[3 − *epi* − 25(*OH*)*D*3]

**Table 7:** Kinetic equations for the reduced vitamin D metabolism model network depicted in Fig. 2. Reaction terms *R,* for *i* = 1, …8 correspond to the respective rate equations as depicted in Table 6. Concentrations are for the vitamin D metabolites 25-hydroxyvitamin D3 (25(OH)D3); C3-epimer 25-hydroxyvitamin D3 (3-epi-25(OH)D3); 1,25-dihydroxyvitamin D3 (1,25(OH)_2_D3) and 24,25-dihydroxyvitamin D3 (24,25(OH)_2_D3).

*d* [25(*OH*)*D*3]/*dt*	= *R*_1_ − *R*_2_ − *R*_3_ − *R*_4_ − *R*_5_
*d* [1,25(*OH*)_2_*D*3]/*dt*	= *R*_2_ − *R*_7_
*d* [24, 25(*OH*)_2_*D*3]/*dt*	= *R*_4_ − *R*_6_
*d* [3 − *epi* − 25(*OH*)*D*3]/*dt*	= *R*_3_ − *R*_8_

**Table 8:** Steady-state equations for reduced kinetic model of vitamin D metabolism. Relationships between metabolite concentrations at steady-state for the reduced kinetic model of vitamin D metabolism, depicted in Fig. 2 are shown. Metabolites are listed with abbreviations in Table 3. Reaction parameters (*k*_3_ – *k*_8_, *K*, *a*, *P*_25(*OH*)*D*3_) as defined in the rate equations in Table 6. Concentrations and Michaelis constant *K* are measured in nM. Non-catalytic rate constants (*k*_3_ - *k*_8_) are measured in d^−1^. Base production constants (*P_25(OH)D3_*) and maximal enzyme rate (*a*) are measured in nM·d^−1^.

$\begin{array}{c} 0=\;{\left[25(\text{OH})\text{D}3\right]}^{2}\cdot (k_{3}+k_{4}+k_{5})\\ +[25(\text{OH})\text{D}3]\cdot (K\cdot (k_{3}+k_{4}+k_{5})+a-p_{25(\text{OH})\text{D}3})\\ -(K\cdot p_{25(\text{OH})\text{D}3}), \end{array}$	(3.1a)
$[1,25{\left(\text{OH}\right)}_{2}\text{D}3]=\frac{a}{k_{7}}\cdot \frac{\left[25(\text{OH})\text{D}3\right]}{K+[25(\text{OH})\text{D}3]},$	(3.1b)
$\left[24,25{\left(\text{OH}\right)}_{2}\text{D}3]=\frac{k_{4}}{k_{6}}\cdot [25(\text{OH})\text{D}3\right]$	(3.1c)
$\left[3-\;\text{epi}\;-25(\text{OH})\text{D}3]=\frac{k_{3}}{k_{8}}\cdot [25(\text{OH})\text{D}3\right]$	(3.1d)

**Table 9:** Summary of vitamin D metabolite ratios from the reduced kinetic model. Metabolite ratios defined from the reduced kinetic model (Fig. 2) using steady state equations (Table 8). Michaelis constants *K* are shown in nM and estimated value shown in Supplementary Table 4. Metabolites are listed with abbreviations in Table 3.

$\alpha =\frac{\left[1,25{\left(\text{OH}\right)}_{2}\text{D}3\right]}{\left[25(\text{OH})\text{D}3\right]}\cdot (K+[25(\text{OH})\text{D}3])$	(1α-hydroxylation)	(3.2a)
$\beta =\frac{\left[3-epi-25(OH)D3\right]}{\left[25(\text{OH})D3\right]},$	(epimerisation)	(3.2b)
$\gamma =\frac{\left[24,25{\left(\text{OH}\right)}_{2}\text{D}3\right]}{\left[25(\text{OH})\text{D}3\right]}$	(24-hydroxylation)	(3.2c)
