# Supplementary material for: Defining vitamin D status using multi-metabolite mathematical modelling: A pregnancy perspective
Source: J Steroid Biochem Mol Biol. Author manuscript; Available in PMC 2023 May 15. (PMC7614536; doi:10.1016/j.jsbmb.2019.03.024)
Supplement: Supplementary Material [file EMS175168-supplement-Supplementary_Material.docx]

**Supplemental information**

**Supplemental Table 1. Summary of serum vitamin D metabolite concentrations for the West Midlands cohort.**

Comparison of serum concentrations of 25-hydroxyvitamin D3 (25(OH)D3) nmol/L, 1,25-dihydroxyvitamin D3 (1,25(OH)_2_D3) pmol/L, 3-epi-25(OH)D3 nmol/L, 24,25-dihydroxyvitamin D3 (24,25(OH)_2_D3) nmol/L. Samples groups were: non-pregnant women; first trimester (NP1); third trimester (NP3); pre-eclampsia third trimester (PET) with median and interquartile range (IQR) shown.

|  | **Non-pregnant**  **Median; IQR** | **NP1**  **Median; IQR** | **NP3**  **Median; IQR** | **PET**  **Median; IQR** |
| --- | --- | --- | --- | --- |
| **25(OH)D3 (nmol/L)** | 33.4 (20.8-44.3) | 28.8 (20.3-46.9) | 45.2 (32.5-59.2) | 35.3 (17.7-54.7) |
| **1,25(OH)_2_D3 (pmol/L)** | 34.2 (29.3-55.0) | 113.7 (82.7-198.3) | 254.7 (195.7-310.1) | 171.2 (113.0-236.3) |
| **3-epi-25(OH)D3 (nmol/L)** | 5.1 (3.9-6.4) | 7.6 (6.0-9.2) | 7.5 (5.9-8.6) | 8.8 (5.9-11.8) |
| **24,25(OH)_2_D3 (nmol/L)** | 3.3 (1.6-4.7) | 1.8 (0.8-3.7) | 7.6 (5.6-10.0) | 10.9 (7.3-22.5) |

**Supplemental Table 2. Serum vitamin D metabolite concentrations for the SCOPE cohort of pregnant women at 15 weeks gestation.** Comparison of serum concentrations of 25-hydroxyvitamin D3 (25(OH)D3) nmol/L, 1,25-dihydroxyvitamin D3 (1,25(OH)_2_D3) pmol/L, 3-epi-25(OH)D3 nmol/L, 24,25-dihydroxyvitamin D3 (24,25(OH)_2_D3) nmol/L in healthy normotensive pregnancies (n=25) and prospective pre-eclampsia cases (PET; n=25), with median and interquartile range (IQR) shown.

|  | **Control (n=25)**  **(median; IQR)** | **PET (n=25)**  **Median; (IQR)** |
| --- | --- | --- |
| **25(OH)D3 (nmol/L)** | 44.7 (19.1- 63.5) | 33.1 (20.5-50.8) |
| **1,25(OH)_2_D3 (pmol/L)** | 336.3 (245.5- 508.4) | 388.8 (304.2 – 468.4) |
| **3-epi-25(OH)D3 (nmol/L)** | 2.5 (1.3- 3.7) | 2.6 (1.7- 3.1) |
| **24,25(OH)_2_D3 (nmol/L)** | 6.5 (2.07- 10.7) | 3.2 (1.37- 12.9) |


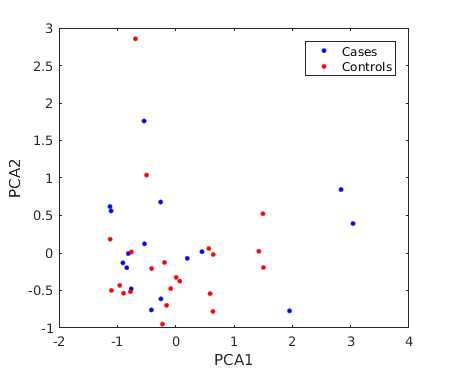


**Supplemental Figure 1. Visualisation of the first two principal components of the normalised SCOPE data.**  Data for figure contains serum vitamin D metabolites for PET and healthy patient serum data from the SCOPE data for all patients where all five metabolites were available (N=16 cases and N=22 controls). Each metabolite value was normalised by multiplying by a scaling factor such that the mean for each normalised metabolite became equal to one (no unit).

**Supplemental Table 3. Major vitamin D metabolite half-life values.** Literature half-life values for 25-hydroxyvitamin D3 (25(OH)D3); C3-epimer 25-hydroxyvitamin D3 (3-epi-25(OH)D3); 1,25-dihydroxyvitamin D3 (1,25(OH)_2_D3) and 24,25-dihydroxyvitamin D3 (24,25(OH)_2_D3).

| Chemical | Half-life |
| --- | --- |
| 25(OH)D3 | 2-3 weeks [31, 32] |
| 1,25(OH)_2_D3 | 4-6 hours [31] |
| 24,25(OH)_2_D3 | 6 days [33, 34] |
| 3-epi-25(OH)D3 | 2-3 weeks [35] |

**Supplemental Table 4. Estimated model parameters for the reduced kinetic model of vitamin D metabolism.** Model parameters for the reduced kinetic model described in **Figure 2, Table 4, and Table 5** were estimated as described in **Supplementary Information**. Metabolites are listed with abbreviations in **Table 3**. We note that *k_7_* captures loss of 1,25(OH)_2_D3 via both 24-hydroxylase and its conversion into 3-epi-1α,25(OH)_2_D3.

| Model parameter | Description | Est. value | Units |
| --- | --- | --- | --- |
| $p_{25(OH)D}$ | Production of 25(OH)D3 | variable | nM•d^-1^ |
| $a$ | Maximum conversion rate of 25(OH)D3 to 1,25(OH)_2_D3 | 0.29 | nM•d^-1^ |
| $K$ | Michaelis constant of conversion of conversion rate of 25(OH)D3 to 1,25(OH)_2_D3 | 47 | nM |
| $k_{3}$ | Epimerisation rate constant of 25(OH)D3 | 0.008 | d^-1^ |
| $k_{4}$ | Conversion rate constant of 25(OH)D3 to 24,25(OH)_2_D3 | 0.01 | d^-1^ |
| $k_{5}$ | Degradation rate constant of 25(OH)D3 | 0.05 | d^-1^ |
| $k_{6}$ | Conversion rate constant of 24,25(OH)_2_D3 | 0.1 | d^-1^ |
| $k_{7}$ | Conversion rate constant of 1,25(OH)_2_D3 | 2.8 | d^-1^ |
| $k_{8}$ | Conversion rate constant of 3-epi-25(OH)D3 | 0.06 | d^-1^ |

**Supplementary Information: Parametrisation of the reduced kinetic mathematical model**

By taking population averages of the vitamin D metabolite data of healthy non-pregnant women from the West-Midlands cohort (**Section 2.1.**), parametrisation of the reduced kinetic model for vitamin D metabolism (**Figure 2, Table 6, Table 7**) was performed. By assuming that measured serum concentrations of vitamin D metabolites are at steady state (i.e. constant), the dynamic ODE model could be reduced to an algebraic system of four equations, as shown in **Table 8**; this is achieved by setting the derivatives in **Table 7** (i.e. the left sides of the equations) to zero. The degradation rate constants *k_6_, k_7_* and *k_8_* were estimated from biological half-life times for vitamin D metabolites (**Supplemental Table 3**). The remaining parameters were then calculated by inputting the relevant metabolite concentrations into the equations in **Table 8** as follows. Parameters *K, k_4_* and *a* follow directly by plugging metabolite concentrations into ((3.1b)-(3.1d), **Table 8**). The quadratic equation (3.1a) was then solved for the positive root which yields an expression of the steady state of 25(OH)D3 in terms of the remaining model parameters $p_{25(OH)D}$ and *k_5_*. These parameters were estimated from the half-life time of 25(OH)D3 in conjunction with the value of 25(OH)D3 at steady state. This is done by fixing the steady state value of 25(OH)D3 to the population average and then iteratively finding the $p_{25(OH)D}$ and *k_5_* such that both (3.1a) holds and the half-life time of 25(OH)D3 is correct by solving the time-dependent ODE system to steady state (**Table 3**). A suitable decay rate for 25(OH)D3 could then be derived, with the rate left undetermined in order to investigate the effect of varying intake of 25(OH)D3 (via diet and/or sunlight). This can be exploited in future studies of the dynamical system. The parametrisation results are tabulate in **Supplemental Table 4**.
